# Effects of FIFA 11 + warm-up program on kinematics and proprioception in adolescent soccer players: a parallel‑group randomized control trial

**DOI:** 10.1038/s41598-023-32774-3

**Published:** 2023-04-04

**Authors:** Mohammadreza Seyedi, Mostafa Zarei, Abdolhamid Daneshjoo, Reza Rajabi, Elham Shirzad, Esmaeil Mozafaripour, Shadan Mohammadpour

**Affiliations:** 1 Department of Sport Medicine, Sport Sciences Research Institute, Tehran, Iran; 2 Sport Rehabilitation and Health Department, Faculty of Sports Sciences and Health, Shahid Beheshti University, Tehran, Iran; 3 Department of Sports Injuries and Corrective Exercises, Faculty of Physical Education and Sport Science, Shahid Bahonar University of Kerman, Kerman, Iran; 4 Department of Health and Sport Medicine, Faculty of Physical Education and Sport Sciences, University of Tehran, Tehran, Iran

**Keywords:** Risk factors, Preventive medicine

## Abstract

This study aimed to compare the effects of 8 weeks 11 + warm-up injury prevention program on kinematics and proprioception in adolescent male and female soccer players. Forty adolescent soccer players (20 males, 20 females) aged between 14–16 years old were randomly assigned into four groups. The experimental group performed the 11 + program for 8 weeks and the control group did their warm-up program. The kinematic variable in a cutting maneuver was measured using VICON motion analysis and ankle and knees’ proprioception by joint position sense (JPS) was measured using a digital inclinometer. For kinematic variables only significant differences in knee valgus among females 11 + compared with female and male control groups were found (*P* < 0.05). Moreover, there were significant improvements in joint position sense variables in 11 + groups compared to control groups (*P* < 0.05). In conclusion, the 11 + program was proven to be a useful warm-up protocol in improving knee valgus and JPS among female and male adolescent soccer players. We suggest adding more training elements to the 11 + program that aimed to enhance the proper alignment of lower extremities which may consequently improve joint kinematics.

## Introduction

Soccer with high demands for dynamic movement, agility, and endurance may exert more loads or forces on the lower extremities joint, which in turn, may raise the frequency of injury^1^. The incidence of injury in soccer is estimated to be high and approximately 1.3 injuries per player per season, with most injuries in the lower extremity (87%) and resulting from noncontact mechanisms (58%)^2^. Specifically, less injury incidence has been associated with superior performance (such as more games won, and more goals scored), players staying on the field instead of off the field, and less injury care cost^3^.

Among the lower extremities injuries risk factors two of the determinant modifiable are the kinematic factors and proprioception. It is has been showed that a less hip flexion angle, smaller amount of knee-flexion joint angle (within 10–30°)^4,5^, and the increased valgus angle at the knee during maneuvers such as jumping-landing or cutting enhance joint loading and injury risks^6^. Furthermore, it a declared that maximum anterior shear force of quadriceps muscles exert when knee-flexion joint angles are within 10 to 30°^7^. In compare to more flexed landing posture, an erect landing posture and small knee and hip flexion angles resultant in higher ground reaction forces (GRF) than a, which finally could lead to a knee injury^8,9^. Joint position sense refers to the accuracy of position replication in different segments, which is an individual’s ability to put their joint angle in a predetermined angle and this is an important for developing functional stability in playing soccer. The central nervous system provides joint position sense from afferent information arising from proprioceptors positioned in the capsules, ligaments, and muscle spindles and use this information for establishing joint stability, postural control, and motor control^9^. Two important predictor risk factors of non-contact anterior cruciate ligament injury are kinematic and joint position sense. Hence, to developing good prevention strategy plan for lower extremities injuries such as ACL injury among soccer players, further research into the modifiable risk factors such as kinematic and joint position sense seems essentials.

Recently, multi-faceted warm-up programs such as the 11 + ^10^, the 11 + Kids^11^, the 11 + S^12^, the HarmoKnee^13^, the Knäkontroll, SISU Idrottsböcker^14^, the neuromuscular training (NMT) program^15^, the bounding exercise program (BEP)^16^ have been implemented as intervention programs to decrease injuries and improve modifiable risk factors among athletes. The widely used warm-up program for the prevention of lower extremity injuries in soccer is the 11 + program. Thompson and their colleagues^17^ performed 15 sessions 11 + and reported it decreased ankle eversion angles during unanticipated cutting among female soccer players. No significant improvement in the 11 + group was found in hip adduction and knee flexion and knee valgus among players aged 10–12 years old^17^. In another study participation in the 11 + during 12 weeks improved hip adduction angle and knee valgus collapse during a 90° cut in the non-dominant leg of collegiate female soccer players. But no significant differences were found in the dominant leg in hip adduction, hip internal rotation, knee abduction, and knee valgus angle^18^. An enhancement in JPS is seen following the 11 + program from pre- to post-test by 2.8% and 1.7% at 45° and 60° knee flexion in the 11 + group but no improvement was reported at 30° knee flexion among male soccer players^10^. Lopes et al. 2019 showed that 10-weeks the 11 + program cannot improve JPS (between 40° and 60° of knee flexion) among male amateur players^19^. Other research reported no significant differences between pre-and post-test in the 11 + group after 6 weeks in the JPS at 45° knee flexion in male amateur soccer players^20^. These conflicting results may arise more need to research in this area.

The need of clarity with respect to the effect of the 11 + injury prevention program on kinematic risk factors such as dynamic knee valgus in male soccer players and conflicting results in this area is important to supply some clear information. There is insufficient information which shows this program is more effective in injury prevention for young male soccer players or female soccer players. Moreover, because of lower extremity injuries consequence such as long-term disability and huge costs on teams and players, identifying effective risk factors such as kinematic variable and JPS and injury prevention factors in the largest sports population in the world (300 million registered players)^21^ can be a critical issue. Therefore, this study aims to investigate the effects of the 11 + warm-up injury prevention program on kinematics and JPS in adolescent male and female soccer players; in a randomized control trial.

## Results

### Kinematics

The one-way ANOVA showed no significant differences among groups in ankle dorsiflexion (F_3,35_ = 1.20, *p* = 0.32), knee flexion (F_3,35_ = 0.88, *p* = 0.46), hip flexion (F_3,35_ = 1.82, *p* = 0.16), trunk flexion (F_3,35_ = 0.60, *p* = 0.62) and trunk lateral flexion (F_3,35_ = 2.17, *p* = 0.11). Significant differences among groups in knee valgus were found (F_3,35_ = 4.55, *p* = 0.009, pη^2^ = 0.28). The Scheffe post-hoc showed significant differences between females 11 + with the female control group (*p* = 0.02, d = 1.05) and the male control group (*p* = 0.03, d = 0.73). No differences between male 11 + with the male control group (*p* = 0.77), male 11 + with female 11 + (*p* = 0.24), male 11 + with the female control group (*p* = 0.67), and male control with female control (*p* = 0.99) were found.

Paired sample *t* test showed significant differences in female 11 + group in ankle dorsiflexion (t = 2.57, *p* = 0.03, d = 0.85) by 21.3%, knee flexion (t = 3.16, *p* = 0.01, d = 1.05) by 6.6%, knee valgus (t = 4.85, *p* = 0.001, d = 1.62) by 57.9%, hip flexion (t = 2.78, *p* = 0.02, d = 0.93) by 14% and trunk lateral flexion (t = 3.18, *p* = 0.01, d = 1.06) by 15.8%. There were no significant differences between pre-test and post-test in all other variables of groups (*p* > 0.05) (Table 1).

**Table 1:** Kinematic variables (degrees) (values are mean ± *SD*), and percentage of change (∆%) (values are (post-test-pre-test/pre-test) × 100).

	Male				Female			
	Pre-test	Post-test	*t* test, *p* value	∆%	Pre-test	Post-test	*t* test, *p* value	∆%
The 11 + group								
Ankle dorsiflexion	29.8 ± 7.3	30.2 ± 5.1	t = 0.14, *p* = 0.89	1.3	19.2 ± 5.6	23.3 ± 5.8*	t = 2.57, *p* = 0.03	21.3
Knee flexion	52.4 ± 5.3	53.4 ± 5.4	t = 0.56, *p* = 0.59	1.9	45.6 ± 5.4	48.6 ± 3.7*	t = 3.16, *p* = 0.01	6.6
Knee valgus	10.5 ± 6.3	8.2 ± 2.8	t = 1.16, *p* = 0.28	21.9	12.6 ± 5.1	5.3 ± 5.1*^,a^	t = 4.85, *p* = 0.001	57.9
Hip flexion	51.2 ± 7.1	51.5 ± 5.8	t = 0.14, *p* = 0.89	0.6	38.6 ± 9.0	44.0 ± 8.8*	t = 2.78, *p* = 0.02	14.0
Trunk flexion	5.8 ± 4.7	7.7 ± 3.5	t = 1.04, *p* = 0.32	32.7	5.5 ± 2.2	7.4 ± 3.5	t = 1.34, *p* = 0.22	34.5
Trunk lateral flexion	10.5 ± 4.0	8.4 ± 2.8	t = 2.08, *p* = 0.07	20.0	13.3 ± 2.9	11.2 ± 1.7*	t = 3.18, *p* = 0.01	15.8
Control group								
Ankle dorsiflexion	24.2 ± 10.6	25.0 ± 7.9	t = 0.53, *p* = 0.61	3.3	22.4 ± 3.0	21.6 ± 3.2	t = 0.76, *p* = 0.47	3.6
Knee flexion	54.1 ± 7.3	54.6 ± 7.9	t = 0.39, *p* = 0.70	0.9	52.1 ± 6.4	51.4 ± 5.8	t = 0.32, *p* = 0.75	1.3
Knee valgus	8.2 ± 6.4	8.3 ± 2.7	t = 0.09, *p* = 0.93	1.2	9.4 ± 4.9	9.9 ± 3.5	t = 0.35, *p* = 0.74	5.3
Hip flexion	41.2 ± 3.1	42.1 ± 4.4	t = 0.53, *p* = 0.61	2.1	37.5 ± 5.9	38.6 ± 6.5	t = 1.57, *p* = 0.15	2.9
Trunk flexion	5.1 ± 3.1	5.4 ± 2.2	t = 0.41, *p* = 0.69	5.9	4.5 ± 2.1	4.6 ± 2.6	t = 0.24, *p* = 0.82	2.2
Trunk lateral flexion	10.5 ± 2.6	10.1 ± 3.2	t = 0.94, *p* = 0.37	3.8	11.4 ± 2.7	11.7 ± 3.5	t = 0.31, *p* = 0.76	2.6

*SD* standard deviation, *t test* paired sample *t* test.

*Significant differences between pre-test and post-test.

### JPS

For the knee, large magnitude area differences with the knee JPS at 3000B0 (F_3,35_ = 20.65, *p* = 0.001, pη^2^ = 0.64) with male 11 + exhibiting less overall JPS error after intervention than male control (*p* = 0.001, d = 2.62) and female control (*p* = 0.04, d = 1.45) groups. Moreover, less JPS error in post-test in the female 11 + group compared with female control (*p* = 0.001, d = 2.31) and male control (*p* = 0.001, d = 3.48) groups was found. Significant JPS difference among groups at 45° knee flexion (F_3,35_ = 9.44, *p* = 0.001, pη^2^ = 0.45) revealed less JPS error with male 11 + over male control (*p* = 0.009,d = 1.57). The Female 11 + group showed less JPS error compared with the female (*p* = 0.01, d = 1.81) and male (*p* = 0.001, d = 1.64) control groups. With the knee, JPS at 60° flexion (F_3,35_ = 6.93, *p* = 0.001, pη^2^ = 0.38) males 11 + showed less error with male control (*p* = 0.01, d = 1.42) while female 11 + group showed less JPS error compared with male control (*p* = 0.002, d = 1.64) group (Table 2).

**Table 2:** Knee and ankle proprioception (degrees) (values are mean ± *SD*), and percentage of change (∆%) (values are (pre-test-post-test/post-test) × 100).

	Male				Female			
	Pre-test	Post-test	*t* test, *p* value, Cohens’ d	∆%	Pre-test	Post-test	*t* test, *p* value, Cohens’ d	∆%
The 11 + group								
Ankle Pl.Fl 20°	5.3 ± 2.1	2.4 ± 1.3	t = 3.02, *p* = 0.01, d = 0.96	120.8	2.5 ± 1.5	1.2 ± 0.6	t = 2.00, *p* = 0.08, d = 0.67	108.3
Ankle Do.Fl 20°	4.8 ± 2.3	2.8 ± 1.4	t = 2.45, *p* = 0.04, d = 0.78	71.4	3.0 ± 1.8	1.5 ± 0.7	t = 2.38, *p* = 0.04, d = 0.79	100.0
Knee 30°	4.4 ± 3.1	2.2 ± 0.9	t = 2.34, *p* = 0.04, d = 0.74	100.0	4.4 ± 2.4	1.3 ± 0.7	t = 3.46, *p* = 0.009, d = 1.15	238.5
Knee 45°	6.5 ± 3.1	2.3 ± 0.8	t = 3.73, *p* = 0.005, d = 1.18	182.6	3.6 ± 2.0	1.7 ± 0.8	t = 3.24, *p* = 0.01, d = 1.10	111.8
Knee 60°	4.9 ± 1.9	2.2 ± 0.9	t = 4.83, *p* = 0.001, d = 1.53	122.7	3.2 ± 1.8	1.8 ± 1.0	t = 3.30, *p* = 0.01, d = 1.10	77.8
Control group								
Ankle Pl.Fl 20°	4.4 ± 2.4	4.8 ± 2.2	t = 0.98, *p* = 0.35	8.3	3.4 ± 1.6	3.5 ± 0.9	t = 0.05, *p* = 0.96	2.8
Ankle Do.Fl 20°	3.7 ± 1.2	3.6 ± 1.1	t = 0.15, *p* = 0.88	2.8	3.5 ± 1.3	3.2 ± 0.9	t = 0.66, *p* = 0.52	9.4
Knee 30°	4.9 ± 2.4	5.6 ± 1.6	t = 0.85, *p* = 0.42	12.5	4.0 ± 2.6	4.0 ± 1.5	t = 0.05, *p* = 0.96	0.0
Knee 45°	4.2 ± 2.0	4.7 ± 2.0	t = 0.85, *p* = 0.42	10.6	3.3 ± 2.0	4.1 ± 1.7	t = 1.31, *p* = 0.22	19.5
Knee 60°	4.2 ± 2.2	4.5 ± 2.1	t = 0.31, *p* = 0.76	6.7	3.4 ± 2.1	2.9 ± 1.1	t = 0.75, *p* = 0.47	17.2

*Do.Fl* dorsiflexion, *Pl.Fl* plantar flexion, *SD* standard deviation, *t test* paired sample *t* test.

*significant differences between pre-test and post-test.

For ankle at 20° plantar flexion (F_3,35_ = 11.19, *p* = 0.001, pη^2^ = 0.49) significantly less JPS error was revealed in males 11 + with the male control group (*p* = 0.006, d = 1.33). Results showed less JPS error in the female 11 + group over the female (*p* = 0.02, d = 3.01) and male (*p* = 0.001, d = 2.23) control groups. In the ankle, at 20° dorsiflexion (F_3,35_ = 6.65, *p* = 0.001, pη^2^ = 0.36) less JPS error was exhibited in the female 11 + group over female (*p* = 0.02, d = 2.11) and male (*p* = 0.002, d = 2.28) control groups (Table 2).

## Discussion

In this study, we aimed to investigate the effects of 8 weeks of FIFA 11 + warm-up injury prevention program on knee kinematics and knee and ankle joint position sense in adolescent male and female soccer players. The main finding was that 8 weeks of FIFA 11 + warm-up program has the potential to improve knee valgus angle which has been considered as the main predicted angle for ACL injuries.

The finding of the current study showed among all kinematic factors the knee valgus angle in both gender significantly improved. Knee valgus is a faulty movement pattern characterized by the tibial and femoral motions resulting in the medial displacement of the knee joint^22^. Cochrane et al. (2010) in a study investigate the effectiveness of 4 training programs (machine weights, free weights, balance training, and machine weights + balance training on kinematic factors of different sports athletes. The result of this study showed knee valgus in the balance training group and weights training group significantly decreased^23^. These results indicate the effectiveness of resistance training and balance training on knee valgus. Exercise number 5 and 10 in FIFA 11 + warm-up program is especially considered balance and distribution exercises, which in an advanced form of them, with the progress of the exercise, more balance challenge by the training partner or throwing and receiving the ball add to them. Moreover, several exercises (exercise number: 5, 6, 9, 11–14) in the FIFA 11 + program are resistance exercises and are designed for lower extremity muscle strengthening. Therefore, according to these cases, probably part of the improvement in knee valgus of current study subjects was due to the improvement of movement control strategy and balance ability. On the other hand, some studies indicate the positive effect of plyometric exercise on movement control such as knee valgus^24,25^ they state this kind of exercise by increasing muscles feedforward activations and co-contraction can improve neuromuscular movement control^24,25^.In the FIFA 11 + program exercise number 12 in all three levels of difficulty and exercise number 14 are two specific plyometric exercises in this protocol. May this exercise by improvement in above mention factor influence the knee valgus in the current study. Moreover, in a study Palmer et al. (2015) examine the effectiveness of two different protocols (isolated hip abductor strengthening group VS functional control group) on knee valgus. Their result showed although the lower extremity muscle strength improved in both groups but the kinematic improvement was only in the functional control group^26^. This result indicates the importance of the neuromuscular aspect of interventions in knee valgus improvement. FIFA 11 + injury prevention program included several exercises, most of which focused on core stabilization, proprioceptive training, dynamic stabilization, and plyometric drills all of them have good potential for neuromuscular adaptations improvement.

Several deficiencies such as anatomical, hormonal, and biomechanical factors cause more prevalence of high knee valgus in women compared with men^27^. It has been suggested that the baseline values of the variables such as knee valgus influenced whether an improvement occurred^28^. on the other hand comparisons of the motion analysis of male athletes in compare with female athletes during a cutting maneuver have indicated that greater knee valgus in female athletes occurred^29^ in line with this, our result showed that adolescent females had greater knee valgus and also our result showed that individuals with more capacity for improvement sustained the greatest changes so female 11 + group showed a significant improvement in knee valgus compared to other groups.

Despite the results related to knee valgus although other kinematics factors showed improvement in both groups but there are no significant differences among the 11 + group and regular warm-up program group in kinematic factors such as Ankle dorsiflexion, Knee flexion, Hip flexion, Trunk flexion, and Trunk lateral flexion. This may be related to the fact which 11 + warm-up program does not address movements in all planes of motion equally^30^. Although dynamic exercises in the 11 + warm-up program such as running drills focus on sagittal plane motions, but the majority of balance and neuromuscular components of the 11 + warm-up program which seems to have more important effects on kinematics variable do address frontal plane knee control. It can be said that this is one of the shortcomings of the 11 + warm-up program includes a low number of unilateral exercises while unilateral tasks and cutting manure are the most important part in soccer.

The second parts of our results indicate 8 weeks of the FIFA 11 + warm-up program significantly improved JPS error compared to the traditional warm-up program. This result confirms that FIFA 11 + warm-up is superior to a regular warm-up program when the aim is to recover proprioceptor acuity of the knee and ankle and hence can positively affect injury prevalence in male soccer players.

In line with the current study Daneshjoo et al. (2012) investigated the effect of the FIFA 11 + warm-up program on knee joint position sense in both genders of football players. The result of their study showed significant improvement in proprioception error of the targeted angle of knee flexion^10^. To our best knowledge, only one study reports inconsistent results with the current study, Lopes et al. (2019) investigate the short and long-term effects of the FIFA 11 + warm-up program on different variables including knee position sense. The result of this study indicates no significant differences between groups in proprioception parameters both in the short and long term^19^. One possible reason for this conflict result may be because their subjects in both groups, showed a low joint position sense error at baseline, possibly making improvement by intervention more difficult. Also, their study was conducted on futsal players in different age groups. The sensorimotor system has different components which involved in maintaining balance and functional joint stability^31^. Poor neuromuscular function can lead to balance and functional joint stability deficiency and different consequent musculoskeletal injuries^32,33^. Exercise interventions such as FIFA 11 + warm-up programs that have balance components may lead to some neural and muscular adaptations which may increase afferent information acuity to the CNS and increase its ability to movement control^34^. These adaptations may be an explanation for the improvement in knee and ankle proprioception which is one of the important elements in the balance component^35^. On the other hand different peripheral receptors such as Muscle spindles, Ruffini endings, Pacini corpuscles, and Golgi tendon organs play a role in the sensation of the joint position around the knee and ankle joints^31^. During the warm-up process tissue temperature, viscoelastic properties, and oxygen delivery to different segments such as knee and ankle joints increased and these changes can lead to enhance above-mentioned mechanoreceptor sensitivity and finally improved joint position sense error^10,36^. Moreover, our result showed adolescent males experience more absolute improvement in joint position senses compared with female adolescence subjects. In this regard Villarreal et al. (2009) report that subjects’ characteristics such as gender can affect response to training adaptation. They reported that men have a greater ability to experience improvements than women^37^ this can be an explanation for better improvement in joint position sense error in adolescence men in compared than adolescence women subject.

There are some limitations in the present study. It has been suggested to achieve a positive and significant effect on biomechanical measures long-term programs should be applied^38^ but the duration of the current study intervention was 8 weeks which may not be enough to make positive changes in all variables. It is possible that the results of this study may not be applicable in other ages group because there is some evidence that training protocols which neuromuscular components such as 11 + warm-up programs are most effective in adolescent populations^39^. Another limitation of this research was that the interventions were applied during the competition season, maybe this condition overshadow the obtained results.

### Clinical implications

Clinicians, researchers, and coach who apply the 11 + warm-up programs as an injury prevention program must consider main effectiveness of this program is on the female adolescent athlete and although for more effectiveness of this intervention some unilateral exercises should be added to this program.

## Conclusion

The main findings of this study were that 11 + warm-up programs have enough potential to improve the JPS and knee valgus in adolescent soccer players and reduce neuromuscular and some biomechanical risk factors during the sport-specific task. Although the result of the current study is in line with previous studies indicating the advantage of 11 + warm-up programs but it seems to better impression on kinematics factors some additional items should be added.

## Methods

### Ethics

The participants were informed orally about the procedures and all of them provided written informed consent. Informed consent from the next of kin, caretakers, or guardians on the behalf of the minors was obtained. The all experimental conditions conformed to the Declaration of Helsinki, and it was approved by the ethical committee of sport sciences research institute of University of Tehran, Iran (Code SSRI.REC-2201–1448).

The Consolidated Standards of Reporting Trials (CONSORT) guidelines were followed to ensure a high quality of reporting. This is a parallel-group randomized controlled trial. A total of 40 participants were selected and randomly assigned into 4 parallel groups. The UMIN Clinical Trials Registry approved the study protocol (Num: UMIN000050048, Date: 24/01/2023).

### Participants

**Figure 1:**
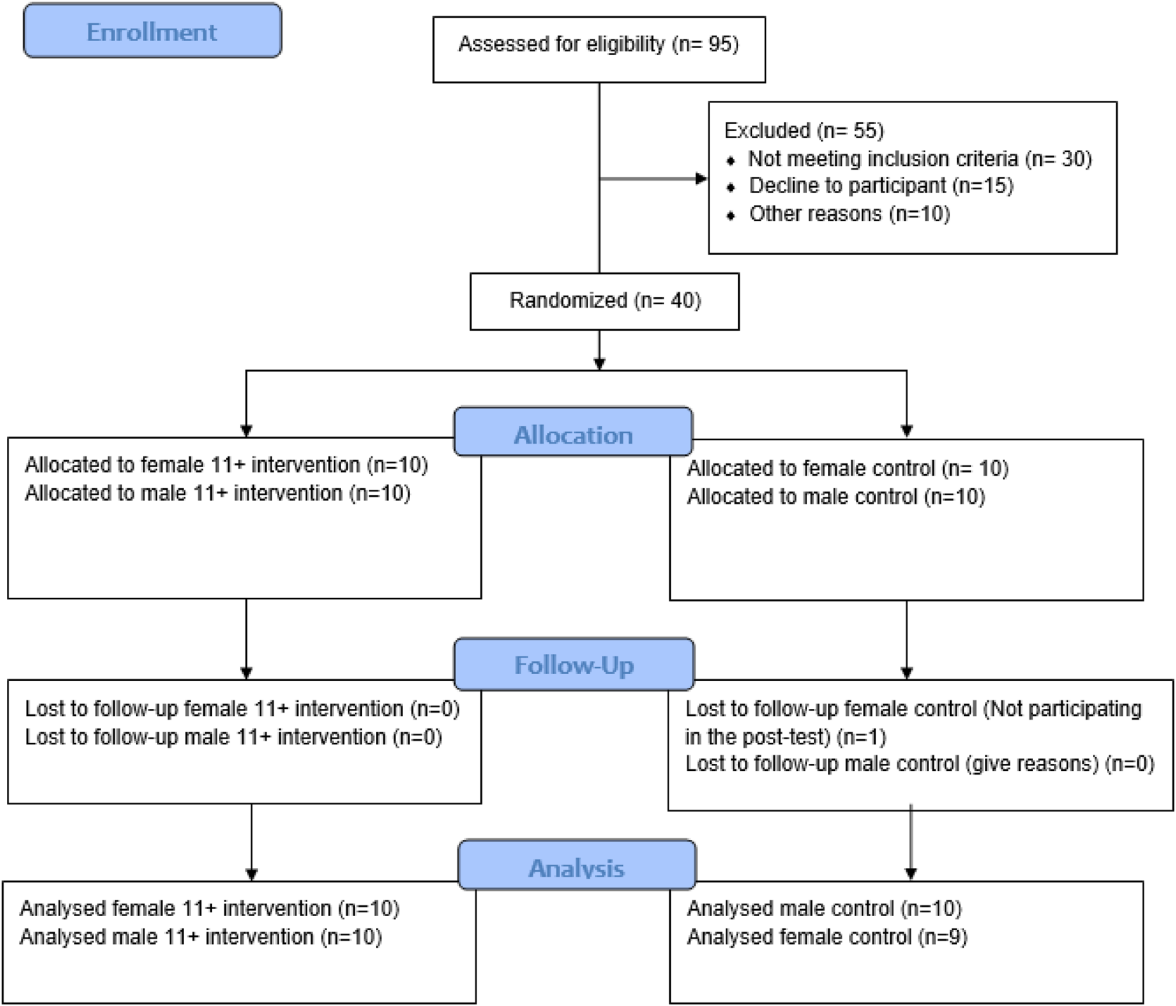
Consort flow chart.

A sample size of 36 soccer player participants was calculated using G*Power software (version3.1.9.2; Kiel, Germany) based on the desired power of 80%, alpha of 0.05, and effect size of 0.6. Finally, forty adolescent professional soccer players with at least 3 years of regular soccer experience were included in the study to consider possible dropouts and randomly assigned to the intervention and control groups^40^. Assignment process is illustrated in the CONSORT diagram (Fig. 1). Demographic characteristics of the participants illustrated in Table 3.

**Table 3:** Demographic characteristics of the participants (value are mean ± SD).

Groups	Male		Female		*P* value
	11 + (n = 10)	Control (n = 10)	11 + (n = 9)	Control (n = 10)	
Age (y)	14.5 ± 0.5	14.2 ± 0.4	14.8 ± 0.7	14.6 ± 0.7	0.279
Height (m)	167.6 ± 5.7	170.5 ± 6.1	165.2 ± 7.1	163.8 ± 4.0	0.076
Mass (kg)	51.5 ± 8.1	53.6 ± 8.7	56.2 ± 6.9	53.6 ± 6.9	0.533

*y* year, *kg* kilogram, *cm* centimeter, *p value* one-way ANOVA results among groups.

The inclusion criteria were age ranged from 14 to 16 years old and they had all experienced at least 3 years’ regular soccer experience playing soccer at Tehran Youth Premier League. These players were employed by their clubs to participate in the premier league. The clubs had almost daily training and played one match per week in a season. The players of one team were randomly selected and assigned to one of the intervention programs. Exclusion criteria consisted of joint laxity (examining the individual Beaton index), neurological, no history of the trunk or lower limb surgery, fractures, and joint replacements of the lower extremity^41^. Goalkeepers were excluded from this study.

### Procedure

The coaches and team managers from the four professional teams were invited to a four-hour instruction course that aimed to introduce the intervention programs. Before starting the intervention program, the coaches and club managers of the 4 groups were invited to an education course that was given by an experienced researcher which aimed to prescribe the warm-up intervention programs in detail (at the mid-season). All the coaches and participants of intervention groups received videos and illustrations of the 11 + program. All the training sessions were supervised and performed by the same study researcher to ensure their compliance with the programs. Verbal encouragement was given throughout the intervention period to motivate the players to concentrate on the quality of their exercise. All tests were conducted between 9:00 a.m. and 1:00 p.m.

### Kinematic tests

Kinematic variables were measured using a Vicon motion analysis system (240 Hz; MX Oxford Metric; Oxford, UK) with 6 cameras (T40s), VICON plug-in gait was used to process motion capture. In order to capture the cutting maneuver, 27 reflective markers (according to Plug-In Gait) were attached to bony landmarks of the lower limb. All Markers were attached with adhesive tape and straps directly to the participant's landmarks. All the markers were attached by the one of researchers. Kinematic data were filtered using a fourth-order zero-lag Butterworth 12-Hz low-pass filter automatically^5^. The dominant leg of each participant was chosen by asking them which leg they would prefer for kicking the ball^42^. The participant performed a general 10-min warm-up with dynamic stretching concentrating on the lower body.

**Figure 2:**
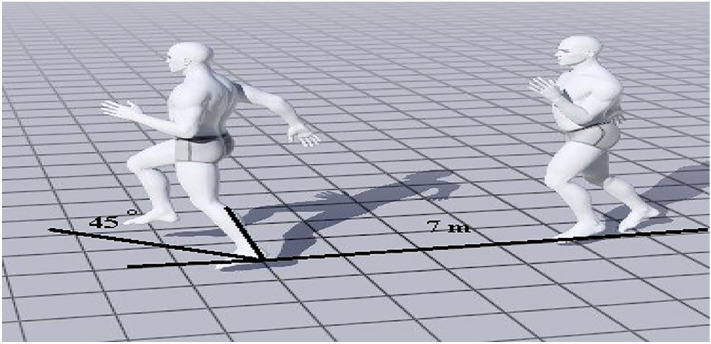
Cutting maneuver from the lateral view (Generated by Magic Poser software version: 1.56.1. https://magicposer.com/").

Before testing, each participant was shown the cutting maneuver and allowed to perform three test trials to familiarize themselves with the actual tests. Participants were allowed to rest for at least 5 min before testing. All participants wore indoor sports shoes. Regarding the cutting maneuver (Fig. 2), the participant was instructed to run fast throughout 7-m until touching with their foot the area of the changing direction, and then perform the cutting. The range between the angles of 35° and 55° with respect to the initial path was found and marked using the marked ground and two cones on the floor, and players were instructed to perform the change of direction at the angle of 45°^43^. Each participant performed three trials with one minute of rest between attempts and the mean of 3 attempts was used for the analysis. Tests were considered acceptable for each participant when there was no deceleration, wrong direction, losing balance, and perform movement patterns other than cutting.

### Joint position sense (JPS)

Knee and ankle JPS were measured by the use of a Limit miniature digital inclinometer (Alingsas, Sweden) by joint repositioning error methods. This method has been widely used and has been reported to have high reliability (ICC = 0.99)^44^. For this purpose, a digital inclinometer was attached to the middle third of the participants' shank of the dominant leg (the preferred leg for kicking a ball) with a hook-and-loop strap. For measuring ankle JPS, a digital inclinometer was attached to the participant's dominant leg foots with a strap The JPS was investigated at a target angle of 30°, 45°, and 60° knee flexion and at 20° of plantar flexion and dorsiflexion of the ankle. The participant was seated on the chair and actively flexed their knee and ankle to the target angle. The subject was asked to move his/her ankle or knee towards the target angle three times with his eyes open, and when he reached this angle, the examiner informed him/her and asked to keep his foot at that angle three times and keep it in his/her mind. Then, to eliminate visual and audible interferences during the measurement, the subject's eyes were blindfolded and wore headphones^10^ and he was asked to actively move his ankle and repositioned the target angle. The participant then held their knee and ankle at the target position for 5 s, and then returned to the starting position. The test was conducted three times, with 30 s of rest in between each test. We analyzed the difference between the target position and reproducing angle by the participants as the joint repositioning error. A lower mean error value indicates a better JPS.

### The FIFA 11 + program

The FIFA 11 + has three parts with a total of 15 different exercises, performed within the indicated sequence at the begin of each training session rather than a regular warm-up program. Part one incorporates slow speed running exercises combined with active stretching and controlled partner contacts, the during part two exercise performed with focusing on core and leg strength, balance, and plyometrics exercise, players perform six sets of each exercises. Every exercise having three levels of increasing difficulty and this program in pat three ending with advanced running exercises (Table 4). The duration of FIFA 11 + in each session is approximately 20–25 min. In the current study the program was performed 3 times per week as a warm-up program for eight weeks under the supervision of the main researcher.

**Table 4:** The ‘‘11 + ’’. Exercises, duration and intensities of the structured warm-up program used (F-MARC).

Exercise	Duration
Section "Introduction": Running Straight ahead, hip out, hip in, circling partner, shoulder contact, quick forward & backwards (6 running items, every items 2 sets)	8 min
Section 2: Strength, plyometric and balance The bench Static, alternate legs and one leg lift and hold (3 items, every items 3 sets)	10 min
Sideways bench Static, raise & lower hip, with leg lift (3 items, 3 sets on each sides)	
Hamstring Beginner (3–5 repetition, 1 set), intermediate (7–10 repetition, 1 set), advanced (12–15 repetition, 1 set). (3 items)	
Single-leg stance Hold the ball, throwing ball with partner, test your partner (3 items, every items 2 sets)	
Squats With toe raise, walking lunges, one-leg squats (3 items, every items 2 sets)	
Jumping Vertical jumps, lateral jumps, box jumps (3 items, every items 2 sets)	
Section 3: Running exercise Across the pitch, bounding, plant & cut (3 items, every items 2 sets)	2 min

### Control group

The control group was asked to use their usual warm-up routine and warm-up without any restrictions and follow their competition schedule.

### Statistical analysis

SPSS software (IBM Corp, Armonk, NY) version 24 was used to analyze data. One-way analysis of variance was used to analyze the anthropometric parameters of the 4 groups at baseline. Shapiro–Wilk test, and Levene’s test were used to determine the normal distribution of data and homogeneity of variance, respectively. After did not confirm the homogeneity of regression slopes assumption of one-way ANCOVA, separate one-way ANOVAs between groups (male 11 + vs. male control vs female 11 + vs. female control) were used for each variable. In the case of statistical significance, the post-hoc Scheffe test was conducted. Effect sizes of each variable were tested using partial eta squared (pη^2^) values (small effect = 0.01, medium effect = 0.06, and large effect = 0.14)^45^. For assessing the kinematic and JPS variables in every group (comparison of pre-test and post-test), the paired *t* test was used. Moreover, the effect sizes for the two independent groups and the dependent *t* tests were tested using Cohen’s d of $\left[ {d = \left( {{{{\text{M1}}{-}{\text{M2}}} \mathord{\left/ {\vphantom {{{\text{M1}}{-}{\text{M2}}} {\sqrt {{{\left( {{\text{SD1}}^{2} \times {\text{SD2}}^{2} } \right)} \mathord{\left/ {\vphantom {{\left( {{\text{SD1}}^{2} \times {\text{SD2}}^{2} } \right)} {2}}} \right. \kern-0pt} {2}}} }}} \right. \kern-0pt} {\sqrt {{{\left( {{\text{SD1}}^{2} \times {\text{SD2}}^{2} } \right)} \mathord{\left/ {\vphantom {{\left( {{\text{SD1}}^{2} \times {\text{SD2}}^{2} } \right)} {2}}} \right. \kern-0pt} {2}}} }}} \right)} \right]$ and $\left[ {{\text{d }} = {\text{ mean}}/{\text{SD}}} \right]$, respectively (with 0.2, 0.5, and 0.8 considered as small, medium, and large effect sizes, respectively).

## Data Availability

The datasets generated during and/or analysed during the current study are available from the corresponding author on reasonable request.
